# 6-BA Reduced Yield Loss under Waterlogging Stress by Regulating the Phenylpropanoid Pathway in Wheat

**DOI:** 10.3390/plants13141991

**Published:** 2024-07-21

**Authors:** Faiza Gulzar, Hongkun Yang, Jiabo Chen, Beenish Hassan, Xiulan Huang, Fangao Qiong

**Affiliations:** 1 State Key Laboratory of Crop Gene Exploration and Utilization in Southwest China, Ministry of Science and Technology, Chengdu 611130, China; faeezagulzar@gmail.com (F.G.); 15354244939@163.com (J.C.); 2 Rice Research Institute, Sichuan Agricultural University, Chengdu 611130, China; beenishhassan2610@gmail.com; 3 Crop Ecophysiology and Cultivation Key Laboratory of Sichuan Province, Sichuan Agricultural University, Chengdu 611130, China; 19130897253@163.com; 4 Key Laboratory of Crop Ecophysiology & Farming System in Southwest China, Ministry of Agriculture and Rural Affairs, Chengdu 611130, China

**Keywords:** 6-BA, IAA, waterlogging, phenylpropanoid, lignin, wheat

## Abstract

Waterlogging stress causes substantial destruction to plant growth and production under climatic fluctuations globally. Plants hormones have been widely explored in numerous crops, displaying an imperative role in crop defense and growth mechanism. However, there is a paucity of research on the subject of plant hormones regulating waterlogging stress responses in wheat crop. In this study, we clarified the role of 6-BA in waterlogging stress through inducing phenylpropanoid biosynthesis in wheat. The application of 6-BA (6-benzyladenine) enhanced the growth and development of wheat plants under waterlogging stress, which was accompanied by reduced electrolyte leakage, high chlorophyll, and soluble sugar content. ROS scavenging was also enhanced by 6-BA, resulting in reduced MDA and H_2_O_2_ accumulation and amplified antioxidant enzyme activities. Additionally, under the effect of 6-BA, the acceleration of lignin content and accumulation in the cell walls of wheat tissues, along with the activation of PAL (phenylalanine ammonia lyase), TAL (tyrosine ammonia lyase), and 4CL (4-hydroxycinnamate CoA ligase) activities and the increase in the level of transcription of the *TaPAL* and *Ta4CL* genes, were observed under waterlogging stress. Also, 6-BA improved the root growth system under waterlogging stress conditions. Further qPCR analysis revealed increased auxin signaling (*TaPR1*) in 6-BA-treated plants under waterlogging stress that was consistent with the induction of endogenous IAA hormone content under waterlogging stress conditions. Here, 6-BA also reduced yield loss, as compared to control plants. Thus, the obtained data suggested that, under the application of 6-BA, phenylpropanoid metabolism (i.e., lignin) was stimulated, playing a significant role in reducing the negative effects of waterlogging stress on yield, as evinced by the improved plant growth parameters.

## 1. Introduction

Wheat is a major cereal crop that commendably contributes to the economic value of countries globally [1]. Universally, the impairment of wheat crop production by climatic fluctuations has appeared as a detrimental factor and a big challenge to food security. Amid abiotic stresses, waterlogging has rigorously hindered and threatened wheat production in Africa, America, and Asia [2,3]. Annually, waterlogging causes approximately 20–50% of the yield loss in wheat crop productivity [4]. Long-term waterlogging conditions always challenge the survival of crops and shift them from growth developmental processes to defensive strategies [5]. Typically, these efforts to survive under waterlogged conditions result in a decline in both the biomass and grain yield of the crop. Typically, these survival efforts tend to result in a reduction of biomass and grain yield in crops.

In order to cope with waterlogging stress conditions for viable wheat production, numerous programs are implemented; for instance, use of growth promoters for the production of waterlogging-stress-tolerant crops [6]. In this respect, there is an imperious need to study the central mechanisms, including physiological responses and metabolic pathways that facilitate important stress responses under waterlogging stress conditions.

To handle the oxygen-deficient conditions induced by waterlogging, plants make many anatomical, physiological, and metabolically adaptive changes, such as the development of adventitious roots and lignin formation and deposition, that increase oxygen availability to the plants [7,8]. Primarily, under stress conditions, plants produce reactive oxygen species scavengers, induction of stomata closure, and osmolyte accumulations to reduce the severe effects of stress [9]. Under waterlogging stress, one of the main strategies of plant is to impair ATP production by shifting from aerobic respiration to anaerobic respiration [10]. In case of long-term waterlogging conditions, a huge quantity of sucrose in wheat partitions to the synthesis of cell wall components, mainly lignin, leading to changes in the cell wall structure. It is, therefore, likely that such modification in the structure of the cell wall helps as one of the tactics used by the plant tissue to reimburse the advanced dissolution of cortical cells for aerenchyma formation, contributing to the preservation of the function of the cell wall under oxygen-deficit conditions [11]. The phenylpropanoid pathway controls lignin biosynthesis under waterlogging stress conditions [12]. In general, the reactions of the phenylpropanoid pathway are catalyzed by PAL (phenylalanine ammonia lyase) and 4CL (coumarate-CoA ligase) [13]. These key regulatory genes, such as *PAL1*, *4CL*, and *C4H*, lead to the biosynthesis of lignin under stress conditions [14]. Lignin accumulation further persuades the signaling response against waterlogging stress [15]. Previously, the waterlogging-induced response of plants has been widely investigated, but further research is needed to determine the intricate wiring of signaling networks. 

Plant hormones play a crucial role in facilitating stress responsiveness through signaling networks [16,17]. Therefore, several plant hormones have been identified as waterlogging-responsive in wheat plant by demonstrating positive or negative associations, such as SA, IAA, JA, cytokinin (CTK), ethylene, and ABA [18,19]. Thus, 6-benzyladenine (6-BA, a synthetic cytokinin) is one of the important plant hormones that regulates many plant developmental processes and mediates plant responses of several abiotic and biotic stresses [20,21]. In case of abiotic stresses, 6-BA plays an imperative role in alleviating the severe effects of cold, drought, salinity, and heat in different plant species, including wheat [21,22]. For example, in maize plant, 6-BA improved waterlogging stress tolerance by improving the antioxidant system of the plant [23]. Consistently, treatment with 6-BA in maize plant improved the plant’s antioxidant activity and reduced H_2_O_2_ content [20]. Under salt stress, 6-BA enhanced photosynthesis, improved endogenous hormone content, and reduced ROS accumulation and the Na concentration in Limonium bicolor [24]. In wheat, exogenous application of 6-BA positively contributed to various morphological parameters, including plant height, flag leaf area, number of tillers, number of grains per spike, and number of spikes per plant [25]. Studies on tea plant and carrot have demonstrated the role of 6-BA in the increase of lignin content under normal growing conditions. For example, in tea plant, 6-BA induced lignin biosynthesis by mediating the expression of CsHCT genes [26]. Further studies on carrot plant showed that 6-BA significantly increased lignin formation by stimulating the lignin biosynthetic genes in the taproots of carrots [27]. Previously, significant insights have been provided about the role of other phytohormones, such as ethylene, ABA, auxin, and jasmonates, in the formation of lignin biosynthesis under normal and waterlogged conditions [12,28]. These studies demonstrated that for these phytohormones, their synergistic/antagonistic interactions are often involved in inducing lignin formation under control or waterlogging conditions. For example, ethylene production led to an increase in the production of lignin content through inducing auxin signaling [29]. Numerous studies also involve the importance of abscisic acid and gibberellins in the regulation of lignin formation. The role of jasmonates has also been detected in the stimulation of lignin formation under normal growth conditions [30], though the aforementioned investigation suggested that these plant hormones do not exhibit a similar role under waterlogging stress conditions. Regardless of these studies, the function of 6-BA in modifying the adaptable physiological and metabolic responses under waterlogging conditions is not properly understood in wheat crop.

To gain insights into the function of 6-BA in mediating morphological, physiological, and metabolic modification to control yield loss of wheat under waterlogging stress, this research examines the effect of exogenous 6-BA on yield loss through the formation of phenylpropanoids (lignin) in waterlogged wheat plants, and further explores changes in the enzyme activity and expression patterns of phenylpropanoid biosynthesis genes along with endogenous hormone levels that produce lignin in response to waterlogging. 

## 2. Results

### 2.1. 6-BA Improved Wheat Morphology under Waterlogging Stress

**Figure 1:**
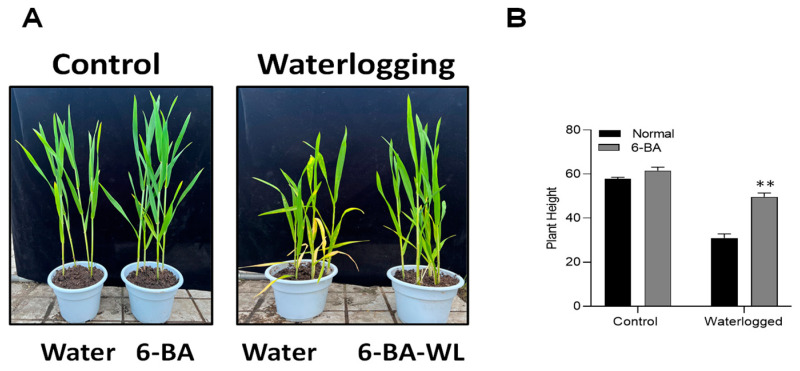
Phenotype of wheat plants under waterlogging stress and 6-BA treatment: (**A**) Wheat plants (6-BA application) were treated with waterlogging treatment for 14 days. (**B**) The plant height of wheat seedlings under waterlogging stress. Significant differences compared to the control are denoted by asterisks (Student’s *t*-test, ** *p* < 0.01). The error bars represent the standard error (SE), with 3 replications.

To confirm the function of 6-BA in the waterlogging stress response, 6-BA was exogenously applied (30 mg/L) on the wheat plants. About 35-day-old wheat seedlings were used for waterlogging and control treatments. Under both conditions, 6-BA-treated plants displayed improved plant growth, whereas the non-treated waterlogged seedlings were more affected than the 6-BA-treated seedlings under waterlogged conditions (Figure 1A). 

Under waterlogging treatment, the height of 6-BA-treated plants was significantly higher than non-treated plants (Figure 1B). These results indicated the positive association of 6-BA application with waterlogging stress tolerance in wheat plants. 

### 2.2. 6-BA Enhanced the Physiological Response of Wheat under Waterlogging Stress

**Figure 2:**
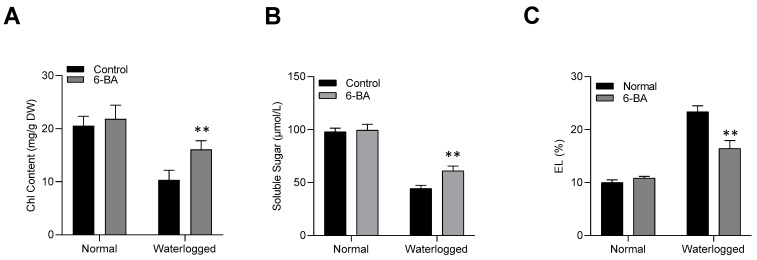
6-BA enhanced the physiological response of wheat under waterlogging stress: (**A**) Chlorophyll content, (**B**) Soluble sugar contents, and (**C**) Electrolyte leakage of 6-BA-treated and non-treated seedlings under waterlogging and control conditions. Significant differences compared to the control are denoted by asterisks (Student’s *t*-test, ** *p* < 0.01). The error bars represent the standard error (SE), with 3 replications.

Waterlogging stress conditions hinder plant photosynthesis by negatively affecting chlorophyll content. To assess the role of 6-BA in waterlogging stress tolerance, we measured the chlorophyll contents in both control and stressed plants. Under waterlogging stress, 6-BA-treated plants exhibited significantly higher chlorophyll content compared to non-treated plants. However, under control conditions, no significant difference in chlorophyll content was observed between treated and non-treated plants (Figure 2A). Waterlogging stress also reduced the storage of soluble sugar in the leaf of the main stem [31]. We also observed decreased soluble sugar content under waterlogging stress, as compared to control plants, but the exogenous application of 6-BA under waterlogging stress showed a higher amount of sugar compared to the non-treated plants (Figure 2B). In the case of control conditions, the difference was not significant for both treated and non-treated seedlings. Additionally, the leaves of non-treated plants displayed significantly higher electrolyte leakage than 6-BA-treated plants (Figure 2C). These data further substantiate the waterlogging tolerance through the exogenous application of 6-BA in wheat plants.

### 2.3. 6-BA Decreased ROS Accumulation through Increasing Antioxidant Enzyme Activity under Waterlogging Stress

Under waterlogging stress, the overproduction of reactive oxygen species (H_2_O_2_) causes oxidative injury in plants [32]. To evaluate the function of 6-BA in response to oxidative pressure, the activity of H_2_O_2_ was examined under waterlogging stress in plants. 

**Figure 3:**
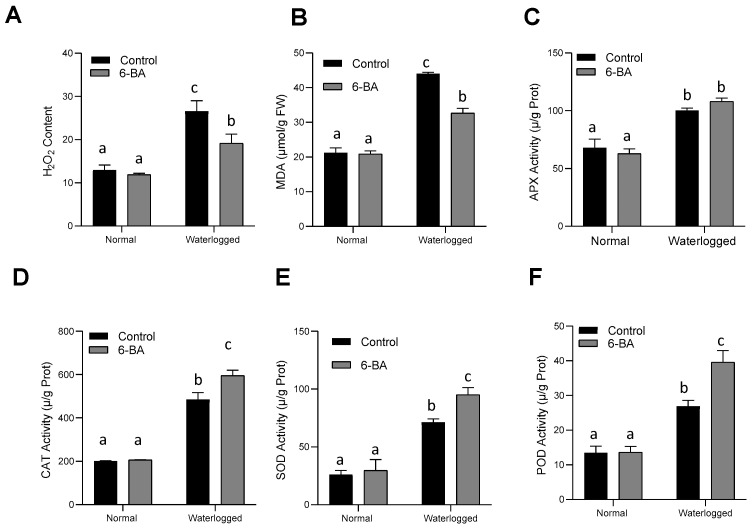
6-BA decreased ROS accumulation through increasing antioxidant activity under waterlogging stress: (**A**) H_2_O_2_ content, (**B**) MDA content, and antioxidant enzyme activity for APX (**C**), CAT (**D**), SOD (**E**), and POD (**F**) of 6-BA-treated and non-treated wheat seedlings under waterlogging stress and control conditions. Significant differences are represented by different lowercase letters (LSD test, *p* < 0.05). The error bars indicate the standard error (SE), with 3 replications.

After waterlogging treatment for 14 days, 6-BA accumulated less H_2_O_2_, as compared to most of the non-treated plants. In the meantime, no significant difference was observed between the control and 6-BA-treated seedlings under control conditions (Figure 3A). Additionally, membrane lipid damage (MDA) through excessive oxidative damage was also caused by waterlogging stress [33]. Therefore, we investigated membrane lipid damage in both control and 6-BA-treated plants subjected to waterlogging stress. Significantly less MDA production in 6BA-treated plants as compared to non-treated plants was observed under waterlogging stress (Figure 3B), signifying less membrane damage by treatment with 6-BA since antioxidant enzymes, such as CAT, APX, POD, and SOD, are involved in the ROS-scavenging system to defend plants from oxidative injury. In this study, we investigated the activity of POD, CAT, APX, and SOD in the 6-BA-treated and non-treated plants under normal and waterlogged conditions. As shown in Figure 3, the antioxidant enzyme activity of 6-BA-treated plants was significantly higher than that of the non-treated plants under stress conditions. No dissimilarity was recorded in these activities between the 6-BA-treated and non-treated plants in normal growing conditions (Figure 3C–F). These results suggested that exogenous treatment with 6-BA improved the waterlogging tolerance of wheat plants by accelerating the antioxidant enzyme activities. 

### 2.4. 6-BA Improved Root Growth System under Waterlogging Stress

**Figure 4:**
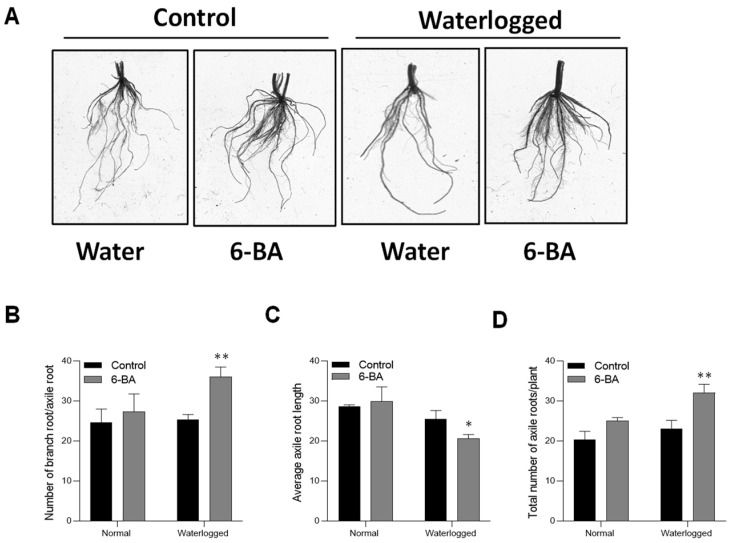
6-BA application improved the wheat root system under waterlogging stress: (**A**) Root growth of 6-BA-treated and non-treated wheat seedlings under waterlogging stress and control conditions. (**B**) Number of branch roots per axile root, (**C**) Average axile root length, and (**D**) Total number of axile roots per plant under waterlogging treatment. Significant differences compared to the control are denoted by asterisks (Student’s *t*-test, * *p* < 0.05, ** *p* < 0.01). The error bars represent the standard error (SE), with 3 replications.

Plants typically modify their root growth in response to waterlogging stress for extra oxygen uptake [34]. We examined root growth modified by 6-BA in wheat plants. During waterlogging stress treatment, the application of 6-BA to plants resulted in a notable increase in the number of axile roots. However, the primary root length experienced a significant decrease under waterlogging stress in the 6-BA-treated plants (Figure 4B,C). In contrast, the non-treated plants exhibited longer roots under waterlogging stress. The 6-BA-treated plants had more than two times the axile roots and branch roots per axile root compared to non-treated plants (Figure 4), signifying higher waterlogging stress tolerance through the shallow root system.

### 2.5. 6-BA Increased Lignin Content and Deposition in the Wheat under Waterlogging Conditions

**Figure 5:**
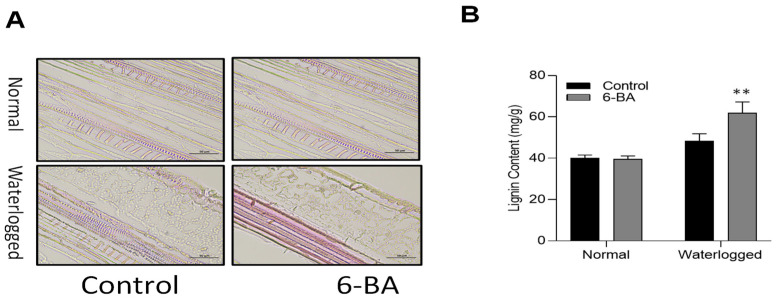
6-BA increased lignin content and deposition in the wheat under waterlogging conditions: (**A**) Visual assessment of lignin deposition in the 6-BA-treated and non-treated wheat seedlings under waterlogging stress and control conditions. (**B**) Lignin content. Significant differences compared to the control are denoted by asterisks (Student’s *t*-test, ** *p* < 0.01). The error bars represent the standard error (SE), with 3 replications.

The exposure of waterlogging stress led to a substantial effect on the lignin content of the wheat seedlings [35]. In the present study, we also verified accumulation of lignin in wheat leaves. The results depicted that treatment of wheat seedlings with 6-BA augmented the lignification of wheat plants compared to the non-treated plants under waterlogging stress (Figure 5A). These results were further confirmed by the quantification of the lignin content under waterlogging stress. Wheat seedlings treated with exogenous 6-BA under waterlogging stress showed higher lignin content, as compared to non-treated plants. The application of 6-BA under normal conditions did not show significant differences in the lignin content. 

### 2.6. 6-BA Contributed to the Phenylpropanoid Pathway by Increasing the Related Enzyme Activity under Waterlogging Stress

**Figure 6:**
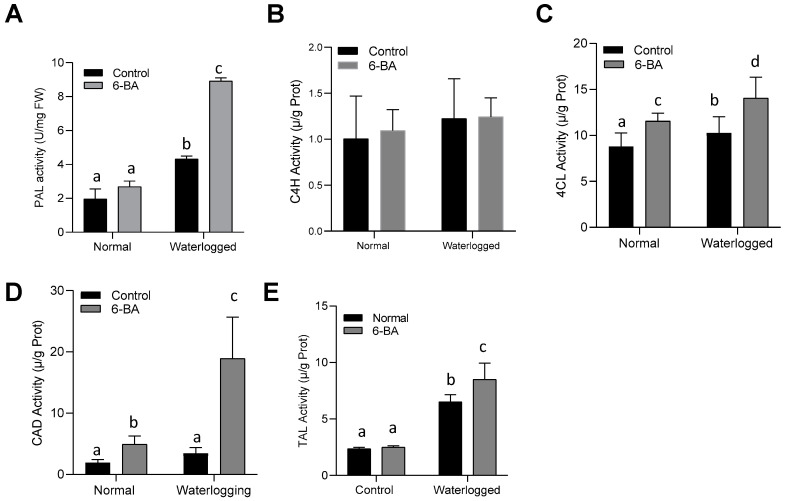
6-BA contributed to the phenylpropanoid pathway by increasing the enzyme activity under waterlogging stress: (**A**) PAL activity, (**B**) C4H activity, (**C**) 4CL activity, (**D**) CAD activity, and (**E**) TAL activity in the 6-BA-treated and non-treated wheat seedlings under waterlogging and control conditions. Significant differences are represented by different lowercase letters, determined through the LSD test (*p* < 0.05). The error bars on the graph indicate the standard error (SE) of three replicates (*n* = 3).

Lignin synthesis is an imperative branch of phenylpropanoid metabolism [36,37]. Usually, PAL (phenylalanine ammonia lyase) is the gateway enzyme of the general phenylpropanoid pathway [38]. First, we investigated the activity of PAL in 6-BA-treated plants under both control and waterlogging conditions (Figure 6). 

The wheat seedlings treated with 6-BA showed significantly higher PAL enzyme activity, as compared to non-treated seedlings. The second step of the general phenylpropanoid pathway is catalyzed by C4H (cinnamic acid 4-hydroxylase) but, in our results, the activity of C4H was not significantly affected by the application of 6-BA. The third step of the general phenylpropanoid pathway is catalyzed by 4CL (4-coumarate-CoA ligase) [39]. The results depicted significantly higher 4CL activity in 6-BA-treated plants, as compared to non-treated plants, under waterlogging stress conditions. The final step of the general phenylpropanoid pathway is catalyzed by CAD (cinnamyl alcohol dehydrogenase). The activity of CAD in 6-BA-treated plants was significantly higher than that in non-treated plants under waterlogging stress conditions. Tyrosine ammonia lyase (TAL) catalyzes the conversion of L-tyrosine to *p*-coumaric acid, a key component in the phenylpropanoid pathway. In the present study, we also observed an increase in the TAL activity by the exogenous application of 6-BA under waterlogging stress conditions. Overall, these results showed that 6-BA enhanced the activity of enzymes involved in the phenylpropanoid pathway under waterlogging stress conditions, thereby promoting lignin biosynthesis and conferring waterlogging tolerance in wheat plants. 

### 2.7. 6-BA Increased the Level of Endogenous Hormone Content under Waterlogging Stress

In order to understand the interplay between 6-BA, phenylpropanoid biosynthesis, and other endogenous plant hormones involved in the regulation of waterlogging tolerance, we conducted an analysis of IAA, ABA, and CTK levels in the shoots and roots of both 6-BA-treated and non-treated plants. This investigation encompassed both normal and waterlogged conditions.

**Figure 7:**
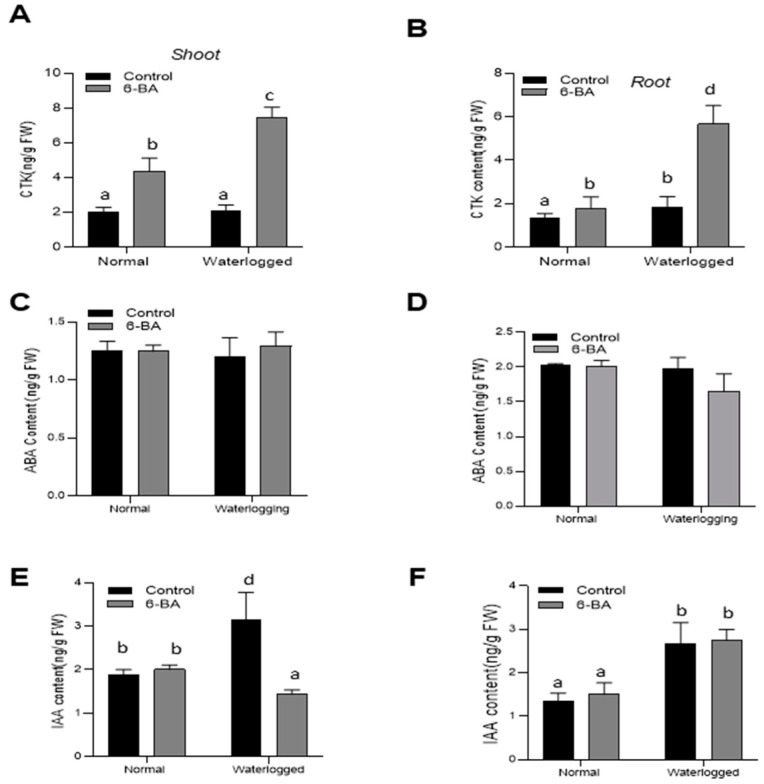
6-BA enhanced the endogenous hormone content in both shoots and roots under waterlogging stress: accumulation of CTK in leaves (**A**) and roots (**B**), accumulation of ABA in leaves (**C**) and roots (**D**), and accumulation of IAA in leaves (**E**) and roots (**F**) under waterlogging stress in 6-BA-treated and non-treated samples. Significant differences are represented by different lowercase letters, determined through the LSD test (*p* < 0.05). The error bars on the graph indicate the standard error (SE) of three replicates (*n* = 3).

Under normal growth condition, no clear differences were observed in the ABA and IAA hormone content of both treated and non-treated plants (Figure 7). In contrast, under waterlogging stress treatment, the plants treated with 6-BA showed a substantial decline in the endogenous IAA content in the shoots of wheat plants, as compared to non-treated plants. Waterlogging, which decreased the lignin content (Figure 7C), led to a significant increase in IAA levels in the non-treated plants. No significant differences were witnessed in the endogenous ABA content, while the level of endogenous CTK was significantly increased in the shoots and roots with the application of 6-BA under waterlogging stress. 

### 2.8. 6-BA Treatment Increased the Expression of Genes Related to IAA Signaling and Phenylpropanoid Biosynthesis

**Figure 8:**
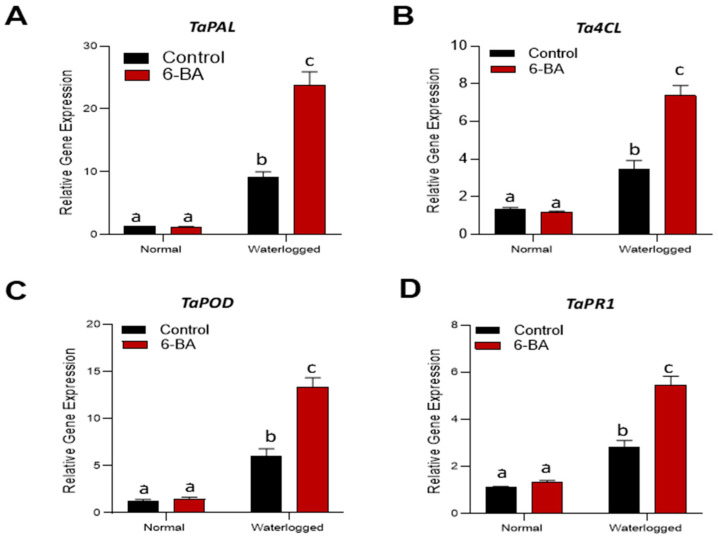
6-BA upregulated the expression of genes associated with antioxidant activity, IAA signaling, and phenylpropanoid biosynthesis pathways. qRT-PCR analysis of 6-BA-regulated genes in 6-BA-treated and non-treated plants under waterlogging treatment and control conditions. (**A**) *TaPAL* and (**B**) *Ta4CL* are involved in phenylpropanoid biosynthesis. (**C**) *TaPOD* is involved in antioxidant activity and (**D**) *TaPR1* is involved in auxin signaling. For reference gene normalization, *TaRL1* was utilized, and the expression level was compared to that of untreated control plants. Statistical analysis using the LSD test (*p* < 0.05) indicated significant differences, as denoted by different lowercase letters. The error bars on the graph represent the standard deviation (SD) of three replicates (*n* = 3).

To obtain a clear picture about the function of 6-BA in the induction of waterlogging stress tolerance through the phenylpropanoid pathway, we examined the influence of 6-BA on the transcriptional activity of the *TaPAL* gene, which encodes the enzyme PAL. *TaPAL* showed increased expression under 6-BA application, as compared to the control plants. Additionally, the qPCR results also revealed that treatment with 6-BA significantly affected the transcriptional activity of the *Ta4CL* gene in the 6-BA-treated seedlings under waterlogging stress (Figure 8). POD activity in the wheat plants under waterlogging stress caused transient activation of the *TaPOD* gene at almost 3-fold higher levels than under non-treated seedlings. At the end, we also checked the expression gene involve in auxin signaling (*TaPR1*), which was significantly higher in the 6-BA-treated plants. Overall, these results clearly validated that 6-BA mediated waterlogging tolerance by increasing phenylpropanoid biosynthesis, activation of antioxidant activity, and auxin signaling.

### 2.9. 6-BA Treatment Decreased the Overall Yield Loss under Waterlogging Stress

**Figure 9:**
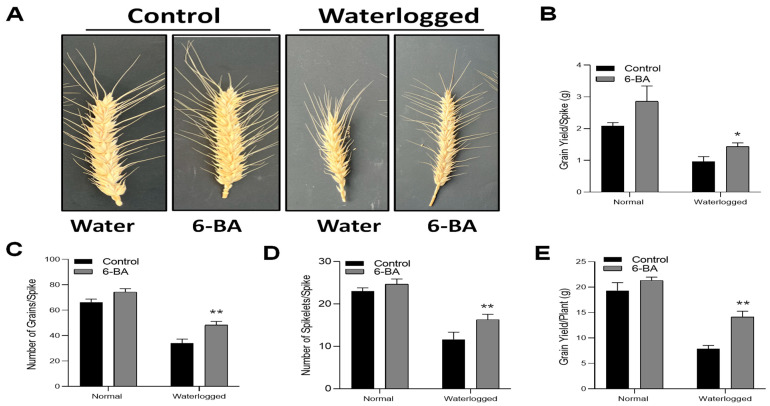
6-BA treatment reduced yield loss under waterlogging stress: (**A**) Photographed spike of wheat with 6-BA-treated and non-treated plants under waterlogging treatment and control conditions. (**B**) Grain yield per spike, (**C**) Number of grains per spike, (**D**) Number of spikelets per spike, and (**E**) Grain yield per plant. Asterisks indicate significant differences (LSD test, * *p* < 0.05, ** *p* < 0.01). Error bars indicate SD (*n* = 3).

Figure 9 shows the effect of 6-BA application on yield and its related components under both control and waterlogged conditions. After waterlogging treatment, the spikes from 6-BA-treated and non-treated plants were observed under both control and waterlogging conditions (Figure 9A). Under control conditions, the spikes did not exhibit any significant differences, while under waterlogging stress, wheat spikes were more affected in control conditions compared to 6-BA-treated plants. Furthermore, we also recorded the grain yield, which was significantly higher in 6-BA-treated plants, as compared to non-treated plants. Number of spikelets per spike, number of grains per spike, and grain yield per plant were higher in 6-BA-treated plants under waterlogging conditions. The results revealed that 6-BA significantly reduced the yield loss by positively maintaining the number of grains per spike and number of spikelets per spike, which led to final yield enhancement under waterlogging conditions.

## 3. Discussion

Wheat, being a vital food and commercial crop, holds great significance in fundamental biological research [40,41]. However, there is a lack of comprehensive knowledge regarding the role of 6-BA in waterlogging stress within the context of wheat. In this study, we aimed to shed light on the beneficial effects of 6-BA in enhancing the tolerance of wheat toward waterlogging stress. We noticed that 6-BA-treated plants exhibited an improved phenotype and morphology under waterlogging stress (Figure 1). Particularly, extra lignin production was detected in 6-BA-treated plants compared to non-treated plants under waterlogging treatment (Figure 5). CTK is an important plant hormone, playing a positive role in the waterlogging stress response to stimulate various physiological processes, including lignin, which is crucial for the cell wall structure under waterlogging stress. Plants treated with 6-BA exhibited higher lignin accumulation and phenylpropanoid-related enzyme activity (Figure 5 and Figure 6), contributing to waterlogging tolerance in the wheat plants. Further, qRT-PCR analysis revealed a higher gene expression of phenylpropanoid-pathway-related genes that are involved in lignin biosynthesis and signaling under waterlogging stress (Figure 8, indicating that 6-BA elevated waterlogging tolerance by regulating lignin synthesis through the phenylpropanoid pathway. 

PAL plays a substantial role in the phenylpropanoid pathway to catalyze the formation of lignin [42]. The earlier studies stated that *PAL* was a key gene in the phenylpropanoid pathway, responsible for the synthesis of lignin, as the *RcPAL* overexpression lines in castor displayed a higher accumulation of lignin content under stress conditions [43]. In the present study, we also detected higher PAL enzyme activity along with an increase in the transcriptional activation of the *TaPAL* gene under waterlogging stress in the 6-BA-treated plants. In the transgenic lines of Arabidopsis plants, *Ta4CL* participated in the lignin deposition, along with plant growth and development [44]. Inhibiting the genetic activity of *Os4CL3* results in a reduction of lignification and thickening of the endothecium, leading to the development of anthers with a wrinkled surface, increased pore size, and condensed length [45]. Moreover, transcriptional activation of phenylpropanoid-pathway-related genes, such as *C4H*, *CAD*, *HCT*, *4CL*, *POD*, and *TAL*, leads to the accumulation of lignin under salt and osmotic stress [37,46]. In the present study, 6-BA also increased 4CL, CAD, and TAL enzyme activity, as well as the gene expression of *TaPAL* and *Ta4CL* in wheat (Figure 7 and Figure 8A), which might demonstrate the involvement of the phenylpropanoid pathway in the waterlogging stress tolerance mechanism in wheat and should be explored in future research. 

Waterlogging stress also leads to an increased production of reactive oxygen species (ROS) due to impaired mitochondrial respiration and heightened activity of plasma-membrane-bound NADPH oxidases. This surge in ROS can cause damage to lipids, proteins, and DNA, resulting in cellular dysfunction and reduced plant vigor. However, phenylpropanoids possess potent antioxidant properties that aid in mitigating the damaging impact of ROS [47]. This property of phenylpropanoids can directly scavenge ROS, protecting cellular components from oxidative damage [48]. In our study, we also detected less H_2_O_2_ content with reduced MDA accumulation in 6-BA-treated plants, as compared to non-treated plants, under waterlogging stress conditions. Additionally, phenylpropanoids also have the ability to regenerate other antioxidants, such as ascorbate and peroxidase, amplifying the plant’s overall antioxidant capacity [49]. Under waterlogging stress, increases in antioxidant enzyme activities, such POD, SOD, and APX, were detected along with increased antioxidant-related gene expression (*TaPOD*) in 6-BA-treated plants (Figure 3 and Figure 8). Our results are consistent with the previous study conducted by Singh [50], who investigated the role of phenylpropanoids in oxidative stress. In this study, our results clearly indicated that 6-BA elevated antioxidant enzyme activity and resulted in less ROS accumulation, which might be a result of activation of the phenylpropanoid pathway. These intricate signaling functions highlight the complexity of phenylpropanoid-mediated stress responses and their crucial role in plants’ adaptation to adverse environmental conditions. Understanding the biosynthesis and regulation of phenylpropanoids under waterlogging conditions can pave the way for developing crop varieties with improved waterlogging tolerance. This advancement has the potential to enhance productivity and sustainability in waterlogged environments, informing breeding programs and biotechnological approaches aimed at enhancing stress resilience in crops. 

Roots play a crucial role in plant function [51], serving as the primary means for water and nutrient uptake and allowing plants to adapt to various environments [52]. Studies have shown that a greater number of axial and lateral roots are associated with improved plant survival under waterlogging stress. In our study, plants treated with 6-BA displayed an increased number of axial roots, but a shorter overall root length compared to non-treated plants (Figure 4). This suggests that the application of 6-BA may lead to the development of a shallower root system in response to waterlogging stress. Previous studies have shed light on the role of plant hormones in stimulating root growth and lignin production under waterlogging stress. For example, IAA has been found to be closely associated with lignin production and its related enzyme activity, promoting lignification [53]. In line with these findings, our research observed a decrease in IAA production in waterlogged plants treated with 6-BA, while non-treated plants showed higher IAA content and less lignin production under waterlogging stress (Figure 7A,B). Interestingly, we noted an upregulation of the auxin signaling gene *TaPR1* in 6-BA-treated plants, suggesting the significance of IAA signaling in regulating lignin production in wheat. Additionally, the enhanced lignin content in 6-BA-treated plants indicated a possible involvement of 6-BA in regulating IAA-mediated lignin accumulation under waterlogging stress conditions. Recent studies have also explored the role of other hormones, such as ABA, in the phenylpropanoid pathway and waterlogging stress response. However, our study found similar levels of ABA in both 6-BA-treated and non-treated plants, regardless of the differences in lignin content (Figure 7B,C). This suggests that ABA may not directly affect the phenylpropanoid pathway and waterlogging stress tolerance in wheat. Overall, these findings indicate that 6-BA influences hormone signal transduction pathways, enabling plants to exhibit faster and stronger defense responses to stress.

Notably, the application of 6-BA reduced the yield loss under waterlogging, as compared to non-treated plants (Figure 9). Importantly, no significant differences were observed in yield, lignin production, hormone content, gene expression, and physiological parameters between the treated and control plants under normal growth conditions. These findings suggest the presence of potential co-factors that may be activated specifically under stress conditions. These co-factors could play a role in fine-tuning the balance between growth and defense mechanisms. Further research is needed to investigate and elucidate these mechanisms in order to gain a deeper understanding of plant responses to waterlogging stress. 

In summary, this study provided valuable insights into the positive effects of 6-BA on waterlogging tolerance, which is attributed to its ability to increase lignin levels through the activation of the phenylpropanoid pathway. This research contributes to our understanding of the complex signaling network involved in waterlogging stress. Through the increment in phenylpropanoids under 6-BA treatment, wheat plants become equipped with a defense mechanism to mitigate the detrimental effects of waterlogging stress, preserving cellular function and maintaining plant vigor. However, further investigation is necessary to uncover additional mechanisms associated with 6-BA-mediated waterlogging tolerance and establish a more comprehensive understanding of the underlying mechanisms. Additionally, future studies should focus on evaluating the performance and reliability of 6-BA application in real field conditions to assess its impact, both positive and negative, on wheat production and waterlogging tolerance.

## 4. Materials and Methods

### 4.1. Experimental Conditions

Wheat variety Shumai 1963 was used in this study. The waterlogging treatment and experimental plant growing were performed according to the Koramutla et al. [54] or 20 min). The pots contained nutrient soil, field soil (collected from the field of Dayi Xiangming Agricultural Company), and vermiculite (15:5:1), along with 18 g of fertilizer (13% NH_4_, 12% P_2_O_5_, and 12% K_2_O). The conditions of the growth chamber were maintained at a photoperiod of 16 h of light followed by 8 h of darkness, along with temperature conditions of 22 °C during the day and 20 °C during the night. The experimental treatments were applied at the 40-day-old stage of the plants. Two distinct treatments were administered: the control group, which was well aerated, and the WL group, which involved submerging the pots. In the case of the WL treatment, the pots were positioned within large containers. For the treatment of waterlogging, every pot was dipped in a new 5 L pot that was already occupied with water. The level of water was maintained 2.5 cm above the soil surface during the whole experiment. More water was added to maintain the water on the soil surface. In the case of controls, plants both with 6-BA treatment and without treatment were used. The non-waterlogged plants were kept without water in 5 L pots. During this whole experiment, the plants were watered with 0.5 L/pot when required. After the waterlogging treatment for 14 days, leaf and root samples were taken from the control and waterlogged plants. Samples were stored at −80 °C for gene expression analysis, enzyme activity, and hormone analysis. Fresh plant tissues samples were used for inspecting the morphological and anatomical parameters considered in the study. Above-ground parts of the waterlogged wheat plants (14 days of waterlogging) and their respective control plants were treated (10 mL/plant) with 30 mg/L of 6-BA (one day before the onset of waterlogging treatment and at three and seven days after the waterlogging). To evaluate the impact of 6-BA under waterlogging stress and control conditions, non-treated plants (sprayed with simple water) were utilized for the control group.

### 4.2. Membrane Lipid Damage (MDA), Antioxidant Enzyme Activity, and Soluble Sugar Content

Membrane lipid damage (MDA) was recorded by using TBA (thiobarbituric acid) as a substrate, which as a result produced thiobarbituric acid–malondialdehyde, which has an absorbance at 532 nm. Antioxidant enzyme activities, comprising POD (peroxide dismutase), SOD (superoxide dismutase), and CAT (catalase), were recorded in 6-BA-treated and non-treated plants using the control and waterlogging samples, as measured previously [55]. To measure the soluble sugar content in leaves of wheat, seedlings were treated with sulfuric acid (98%) and phenol (5%), and then by using a spectrophotometer, the readings of the filtered mixture were measured at 485 nm [56].

### 4.3. Root Growth Measurement

Waterlogging treatment was applied to the 6-BA-treated and non-treated seedlings continuously for 14 days. After 14 days, the roots were collected and cleaned with fresh water for further analysis. First, the roots were photographed, and then length of the roots was recorded. The numbers of axile roots and branch roots per axile root were also counted. Three biological replicates were used for each assay. 

### 4.4. Total Chlorophyll and Electrolyte Leakage Determination

Here, 0.1 g fresh leaf samples were taken from both waterlogged and control plants for the measurement of total chlorophyll (Chl) content. Then, they were added to the dimethyl sulfoxide and kept in the dark for 48 h. The extracts of samples were analyzed photometrically at 663 nm and 645 nm [57]. Quantification of total Chl was performed according to Arnon [58]. The method of electrolyte leakage determination was used as previously described by Hassan, Qi [57], and the final value was estimated following Blum [59]. 

### 4.5. Phenylpropanoid-Pathway-Related Enzyme Activities 

The plant material (2.0 g) was promptly frozen using liquid nitrogen and subsequently stored at a temperature of −80 °C for preservation. Then, the extraction of protein was achieved according to the previous procedures by Jiang, Wang [60]. PAL activity was measured at an absorbance of 290 nm using a spectrophotometer [60]. TAL activity was measured at an absorbance of 310 nm. The activity of 4CL was measured at 333 nm [61], while C4H activity was measured at 340 nm with a spectrophotometer [62]. 

### 4.6. Determination of Lignin Content and Lignin Deposition 

The quantification of lignin content in wheat leaves was carried out by Khadr and Wang [21], employing a validated method. Each sample was analyzed in triplicate to ensure accuracy and reliability of the results. To evaluate the presence of lignin deposition in the cell walls of the wheat leaves, a histochemical approach was employed. Fresh sections of wheat leaves, both treated and untreated with 6-BA for a duration of 14 days, were examined. The intensity of the red–violet lignin staining was visualized using a phloroglucinol-HCl solution [63]. The visualization process involved adding a few drops of a mixture consisting of 5% phloroglucinol in ethanol and 30% HCl solution to the wheat leaf sections. This enabled the detection of lignin through the development of a distinct red–violet color. By following this standardized procedure, the presence and distribution of lignin in the wheat leaves were assessed, providing valuable insights into the impact of the 6-BA treatment on lignin deposition.

### 4.7. Hormone Content Analysis 

To analyze the hormone content, leaves from wheat plants were collected per biological replication after subjecting both 6-BA-treated and non-treated plants to waterlogging treatment. The quantification of understudied hormone contents was performed using the ELISA hormone analysis kit, following the prescribed method.

### 4.8. RNA Extraction and Gene Expression Analysis (qRT-PCR)

Wheat plant leaves per biological replication were collected in liquid nitrogen after waterlogging treatment for both 6-BA-treated and non-treated plants. The RNA extraction process followed the TRI-zol™ Reagent (Tiangen, Beijing, China) manufacturer’s instructions. The integrity of the extracted RNA was confirmed by electrophoresis on 2% agarose gels. cDNA synthesis was performed using the PrimeScript RT Reagent Kit with the gDNA Eraser (TaKaRa Bio, Osaka, Japan). NCBI’s Primer-BLAST program was utilized to design specific quantitative primers, considering parameters such as PCR product length (100–250 bp) and an annealing temperature range of 57–63 °C. The primers and amplified fragments used in this experiment are shown in Table S1. The SYBR GREEN MIX reagent was utilized to check the relative expression of understudied genes on a quantitative PCR machine. The expression levels of the target genes were assessed using the delta–delta Ct method [64]. Each experiment was conducted with three independent biological replicates and three technical replicates to ensure accuracy and reliability.

### 4.9. Grain Yield and Its Related Components

Grain yield was measured from five selected individual plants. All spikes were collected and spikelets were counted and then threshed by hand to measure the total yield per plant. The number of spikelets was counted according to the method in [65].

### 4.10. Statistical Analysis 

We used one-way or two-way ANOVA to measure the statistical significance of differences among the samples. Fisher’s least significant difference test at a significance level of *p* < 0.05 was then conducted to compare the sample means and identify significant variations between them.

## Data Availability

The data supporting the results are presented in the main text and Supplementary Materials.

## Supplementary Materials

The following supporting information can be downloaded at: https://www.mdpi.com/article/10.3390/plants13141991/s1, Table S1: List of primers used in this study.
