# Supplementary material for: 6-BA Reduced Yield Loss under Waterlogging Stress by Regulating the Phenylpropanoid Pathway in Wheat
Source: Plants (Basel). 2024 Jul 21;13(14):1991. doi: 10.3390/plants13141991 (PMC11281113; doi:10.3390/plants13141991)
Supplement: Supplementary file 1 [file plants-13-01991-s001.zip › plants-3083883-supplementary.pdf]

6-BA Reduced Yield Loss under Waterlogging Stress by Regulating the Phenylpropanoid Pathway in Wheat

Faiza Gulzar [1], Hongkun Yang [1,*], Jiabo Chen [1], Beenish Hassan [2], Xiulan Huang [3] and Fangao Qiong [1,3,4,*]

[1] State Key Laboratory of Crop Gene Exploration and Utilization in Southwest China, Ministry of Science and Technology, Chengdu 611130, China; faeezagulzar@gmail.com (F.G.); 15354244939@163.com (J.C.)
[2] Rice Research Institute, Sichuan Agricultural University, Chengdu 611130, China; beenishhassan2610@gmail.com
[3] Crop Ecophysiology and Cultivation Key Laboratory of Sichuan Province, Sichuan Agricultural University, Chengdu 611130, China; 19130897253@163.com
[4] Key Laboratory of Crop Ecophysiology & Farming System in Southwest China, Ministry of Agriculture and Rural Affairs, Chengdu 611130, China
* Correspondence: yhk159357@163.com (H.Y.); fangao20056@126.com (F.Q.)

Table S1: List of primers used in this study

Gene	Forward Primers	Reverse Primers
TaRLI (reference genes)	TTGAGCAACTCATGGACCAG	GCTTTCCAAGGCACAAACAT
TaPAL	GGCGTCAAAACATGGCGTC	AGTCCGAGAAGTCCGAGA
TaPR1	ATAACCTCGGCGTCTTCATC	GCTTATTACGGCATTCCTTTT
TaPOD	TCCGTTGTCGCCTCTGGT	GTGCCTTGCCGATGGTGT
Ta4CL	ACGGTTGGCTGCATACTGG	ATTTCGGCGTGTGTGATGAG
